# Regulation of PDL-1 expression in thyroid carcinoma cells by tumor cell derived cytokines activating STAT3

**DOI:** 10.1007/s12026-024-09552-y

**Published:** 2024-12-19

**Authors:** Matthias Klemke, Nadine Veit, Ingo Schmidt-Wolf, Ralph A. Bundschuh, Markus Essler, Barbara Kreppel

**Affiliations:** 1 Department of Nuclear Medicine, University Hospital Bonn, Venusberg-Campus 1, 53127 Bonn, Germany; 2 Center for Integrated Oncology (CIO), University Hospital Bonn, Venusberg-Campus 1, 53127 Bonn, Germany; 3 Excellence Cluster ImmunoSensation, University Hospital Bonn, Venusberg-Campus 1, 53127 Bonn, Germany

## Introduction

The immune system is an important factor of tumor progression in thyroid cancer. Mutations in oncogens, for example BRAF and Ras, or receptor-tyrosine-kinase fusions activating MAP-kinase and mTOR signaling pathways, cause malignant transformation in thyroid carcinomas and promote malignant growth of mutated thyroid cells [1–3]. Depending of the type of mutation different signal pathways may drive malignant growth leading to the specific types of tumor biology in the different histologic types of thyroid cancer. Typically, the MAP-kinase/ERK pathway is predominant in papillary thyroid cancer. In follicular and anaplastic tumors of the thyroid, PI3-kinase/AKT and mTOR are more dominant [1]. Malignant transformation leads to expression of neo-antigens on the cell surface together with MHC-1, facilitating detection and elimination by immune cells such as NK- and CD8-positive T-cells [4, 5]. Consequently, in thyroid cancer, a number of studies found a close association between immune infiltration and clinical outcome [6, 7]. On the other hand, tumor cells overcome immune control by specific escape strategies [8]. The most important way to overcome immune control is expression of checkpoint molecules such as PD1 on immune- and PDL-1 on tumor-cells blocking anti-tumor immune response. Therefore, PDL-1-expression is an important co-factor of tumor growth [9]. Cytokines such as interferon-γ, Il-8, or IL-6 up-regulate PDL-1 expression in tumor cells by activating the JAK/STAT3 pathway promoting transcription of cytokine response genes [10]. Activation of cytokine receptors induce auto phosphorylation of Tyr705 of STAT3 inducing transcriptional activity. Moreover, Ser727 of STAT3 is substrate to phosphorylation by kinases of the MAP-kinase/ERK family facilitating crosstalk between cytokine receptor- and onocogen-driven signal pathways potentially further enhancing transcription [11, 12]. The main source of these cytokines in thyroid tumors is secretion by immune cells. High expression of PDL1 is present in differentiated, anaplastic, and medullary thyroid cancer together with infiltration by CD4- and CD8-positive T-cells as well as NK cells, secreting cytokines enhancing PDL-1 expression. On the other hand, tumor cells may secrete factors into the microenvironment leading to PDL-1 expression [13, 11, 14–18]. It may also be possible to up- or down-regulate PDL-1 in tumor cells by application of anti-tumor agents or radiation therapy. Elucidation of mechanisms regulating PDL-1 expression in thyroid tumor cells may help to develop novel immunotherapies and improve the understanding of immune-escape mechanisms in thyroid cancer. Therefore, the hypothesis of our study was that thyroid cancer cells actively participate in blocking immune surveillance by secretion of mediators of the innate immune system. We report autocrine secretion of cytokines regulating PDL-1 expression by activating the STAT3 pathway in differentiated and anaplastic thyroid cancer cells.

## Methods

### Cell culture

For cultivating B-CPAP cells RPMI and for CAL-62 cells DMEM culture media containing 10% FCS, 2 mM L-Glutamine and 100 U/mL Pen/Strep was used. Cells were grown at 37 °C in a 5% CO_2_ atmosphere in 75-mL culture flasks (Falcon) with media changes after 3–4 days.

### Preparation of conditioned medium

Cells were incubated with DMEM (CAL-62 cells) or RPMI-1640 (B-CPAP cells) containing 2 mM L-glutamine and 100 U/mL Pen/Strep for 12 h. Supernatants were then collected and diluted 1/2 with fresh medium before use.

### Inhibitors

Interleukin-8 (IL-8), interferon-gamma (IFN-γ), and interleukin-6 (IL-6) were from PeproTech, Cranbury, NJ. GRO-α/CXCL-1 was from Cell Guidance Systems, Cambridge, UK. U0126 was from Promega, WI. MK2206, alpelisib, and sunitinib were from Selleck Chemicals LLC, TX. 5,15-DPP was from Cayman Chemical, MI.

### RT-PCR

For designing PCR primers, we used Primer3 software. Primers were manufactured by Invitrogen/Fischer Scientific, Schwerte, Germany. For PDL-1 two primer pairs were used (P1 (upstream): CTGTCACGGTTCCCAAGGAC, P1 (downstream): CTTGTAGTCGGCACCACCAT and P2 (upstream): ACCTGGCTGCACTAATTGTCT, P2 (downstream): TCCAGATGACTTCGGCCTTG. Two other primer pairs were generated for CTLA4: C1 (upstream): CTGAAGACCTGAACACCGCT, C1 (downstream): ATCATGTAGGTTGCCGCACA and C2 (upstream): GGCACCTCCAGTGGAAATCA, C2 (downstream): TGGGTTCCGCATCCAACTTT. For complimentary DNA (cDNA) preparation, we used the RNeasy® Plus Mini Kit (QIAGEN) according to the manufacturer’s protocol for cell lysis, homogenization, and RNA isolation. A spectrophotometer was used for determination of RNA concentration and integrity. cDNA was then prepared using the SuperScript™ III (Invitrogen) reverse transcriptase protocol: 2 µg of RNA were mixed with 100 ng of random hexamers (Invitrogen) and 10 mM dNTP mix (PeqLab), brought to 12 µL with ultrapure water (Molecular biology grade, Fisher Scientific)**,**and pre-incubated at 65 °C for 5 min. Eight microliters of reaction mix (containing 5 × First Strand Buffer 0.1 M DTT, 40 units RNAseOut, and 200 units Superscript III Reverse Transcriptase) was added and incubated at 50 °C for 60 min to generate cDNA. One microliter of cDNA served as a template for amplification in the actual hot-start polymerase chain reaction using 1 unit HotStarTaq (QIAGEN), 0.5 pmol each up- and downstream primers and 0.2 mM dNTPs. Gel electrophoresis was performed with 10 µL of the amplificate on a 1% peqGold Universal Agarose (PeqLab) in TAE buffer containing 3 µL Midori Green Advanced (Nippon Genetics) per 50 mL gel. Followed by band visualization using an UV transilluminator (Syngene).

### Semiquantitative Western blotting

PD-L1 expression was determined after cells were treated with inhibitors cytokines, lactate, or conditioned medium for the indicated time points with the indicated concentrations. Protein extracts were obtained by cell lysis in ice-cold Triton X-100 solubilization buffer (NaH_2_PO_4_/Na_2_HPO_4_ pH 7.4 containing 1% Triton and protease and phosphatase inhibitors). The protein concentration of each lysate was determined by Bradford assays. Samples were mixed with 5 × Laemmli sample buffer, boiled and loaded on 10% SDS gels. Each lane was loaded with equal amounts of total protein from each sample. After semi-dry transfer to nitrocellulose in blotting (Tris–HCl; 20% methanol) buffer. Membranes were blocked in blocking/incubation buffer (5% milk powder in TBS containing 0.1% Tween-20) for 60 min at room temperature. Primary antibodies (PDL-1 intracellular domain, GAPDH, akt, pAkt (Ser 473), ERK 1/2, pERK1/2 (Thr 202 / Tyr 204), STAT3, pSTAT3 (Ser727), pSTAT3 (Tyr705) were rabbit mAbs from Cell Signaling Technology, Inc., Danvers, MA and diluted 1/1000 as indicated by the manufacturer in the above mentioned blocking/incubation buffer. Membranes were incubated over night with antibody, washed by three times with incubation buffer and incubated for 60 min with peroxidase labeled secondary antibody (Peroxidase-AffiniPure Goat Anti-Rabbit IgG (H + L)—Jackson Immuno Research, West Grove, PA, diluted 1/10,000 in incubation buffer). For development of blots, ECL solution (Super Signal™ West Pico PLUS by Pierce) and an Amersham™ Imager 600 were used. ImageJ (release 1.53r, NIH, Bethesda, MD, USA) and Microsoft Excel (release 2019, Microsoft Corporation, Redmond, USA) were used for semi-quantitative analysis. Statistically significant differences are denoted by *p* < 0.05 (*), and highly significant differences are denoted by *p* < 0.01 (**) and *p* < 0.001 (***), as indicated above the respective error bars. Numerical measurement data are presented as mean ± standard deviation (SD).

### Sandwich enzyme-linked immunosorbent assay (ELISA)

The Proteome Profiler™ Human XL Cytokine Array Kit (R&D Systems) was used to analyze cell culture supernatants for the presence of cytokines according to the manufacturer’s instructions. HRP visualization was detected using the Amersham™ Imager 600 imaging system.

### Lactate determination in conditioned medium

Determination of lactate in the conditioned medium was performed in the central laboratory of the University Hospital Bonn. Lactate is oxidized to pyruvate by the enzyme lactate oxidase. The resulting hydrogen peroxide is then converted by peroxidase into a dye that is directly proportional to the lactate concentration and can be measured by UV/VIS spectroscopy.

## Results

**Fig. 1:**
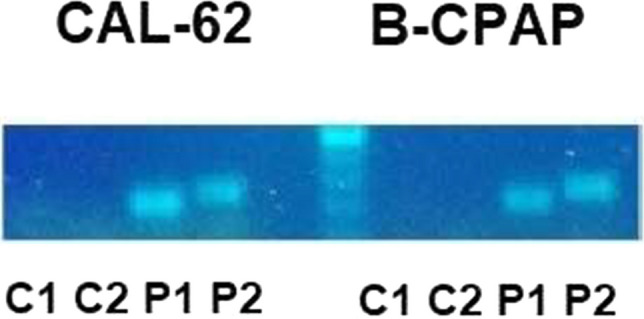
RT-PCR of PDL-1 and CTLA4. Expression of PDL-1 and CTLA4 mRNA was determined using two different primer pairs for each mRNA. C1 and C2 for CTLA4. P1 and P2 for PDL-1. PDL-1mRNA was present in B-CPAP and in CAL-62. Both cell lines were negative for CTLA4

We determined expression of PDL-1 mRNA in the papillary and anaplastic thyroid cancer cell lines BC-PAP and Cal-62 by real time PCR (RT-PCR) using two different primer pairs. CTLA-4 mRNA was used as negative control. PDL-1 mRNA expression is present in both cell types indicating a basal level of transcription in absence of stimulation by immune-cells (Fig. 1).

**Fig. 2:**
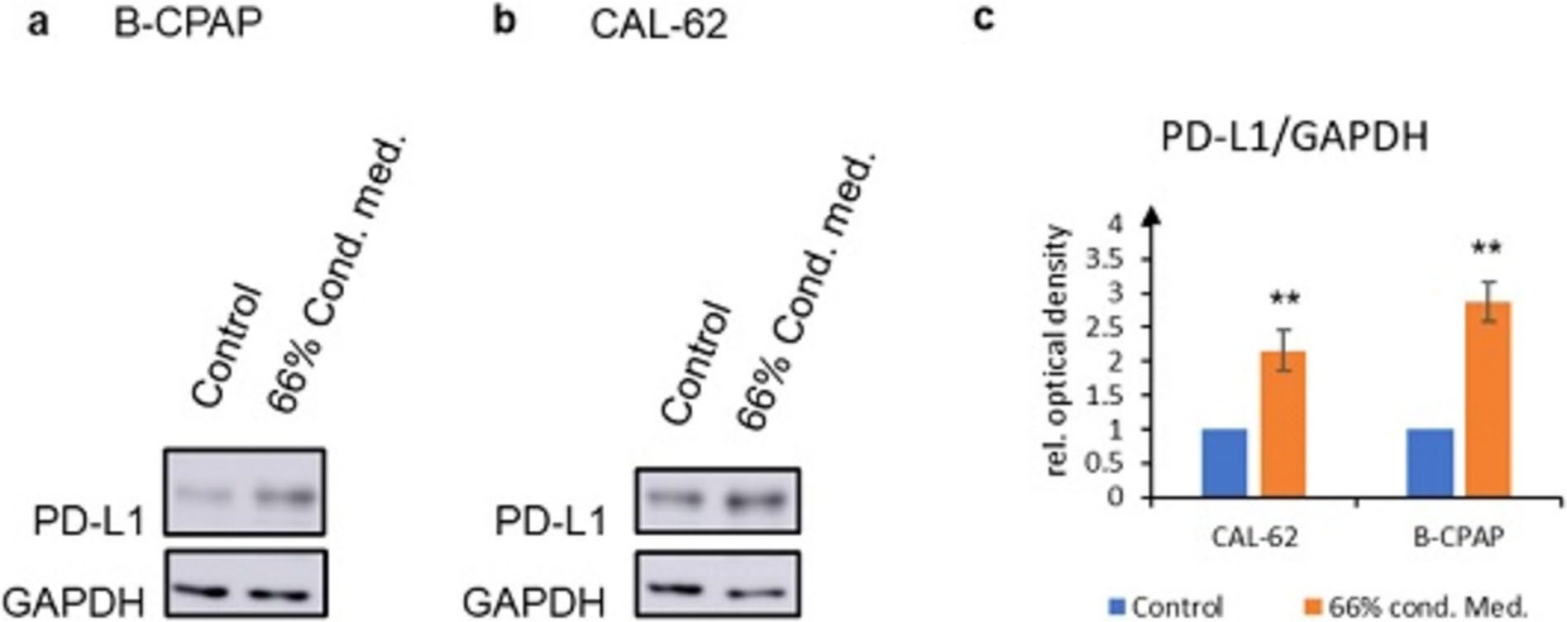
Conditioned medium induces PDL-1 protein expression. After treatment of cells for 72 h with supernatant of the same cell type, PDL-1 expression was determined by Western blot. Representative blots from BC-PAP (**a**) or CAL-62-cells (**b**) are shown. GAPDH was used as loading control. Blots are representative of three independent experiments. **c** Densitometric analysis of PDL-1 expression. The bars represent the mean ± standard deviation from three independent experiments. PDL-1 values were normalized to GAPDH. An unpaired two-tailed *t* test was performed for statistical analysis. **p < 0.01

Next, we studied whether PDL-1 protein expression upon stimulation by autocrine factors secreted by thyroid carcinoma cells [19, 20]. After treatment for 72 h with supernatant of cells of the same type diluted 2:1 with fresh medium, we performed Western Blots. We used BC-PAP or CAL-62 cells treated with fresh medium as controls. A significant increase in PD-L1 expression occurred in both cell lines after treatment with conditioned medium (Fig. 2a, b). Quantification by densitometry showed statistically significant up-regulation of PDL-1 expression (Fig. 2c). These results indicate that agonists up-regulating PDL-1 are present in supernatants of thyroid carcinoma cells.

**Fig. 3:**
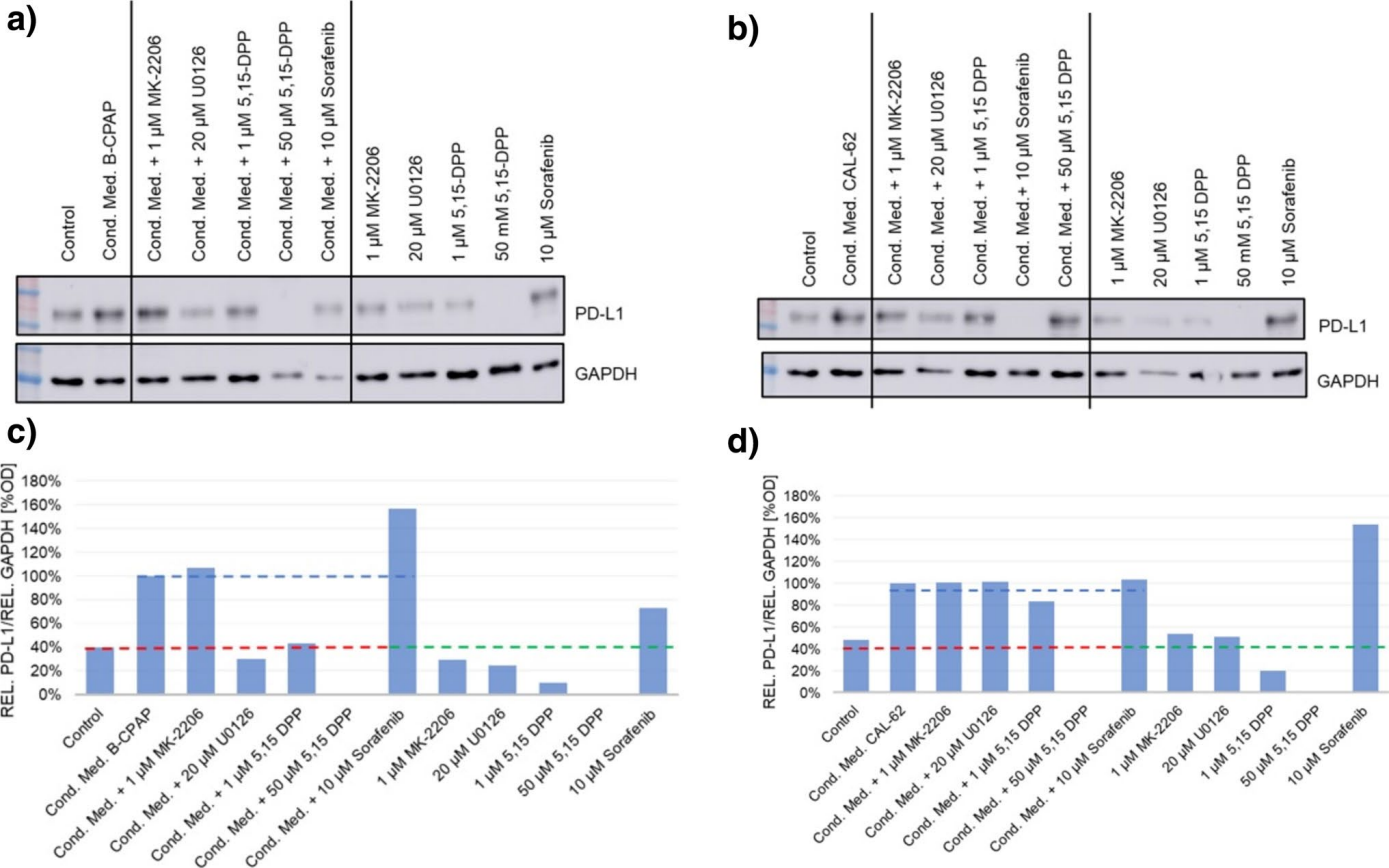
Modulation of PDL-1 by inhibition of STAT3, AKT, and ERK phosphorylation. The figure shows PDL-1 Western blots from cells treated with either normal medium, conditioned medium, pathway inhibitors or combination. **a** B-CPAP cells. **b** CAL-62 cells. Representative Western blots from two independent experiments are shown. Densitometric analysis of PDL-1 Western blots form B-CPAP-cells (**c**) or CAL-62-cells (**d**). The red dashed line indicates the basal level of PDL-1 in control cells. The blue dashed line represents maximal stimulations of PDL-1. The green dashed line represents the basal level of PDL-1. All values were normalized to GAPDH

We analyzed signal pathways mediating up-regulation of PDL-1 in thyroid carcinoma cells after stimulation with conditioned medium and pathways regulating PDL-1 expression in un-stimulated thyroid carcinoma cells. For this purpose, we used specific inhibitors of STAT3-, PI3K-AKT-, and ERK-pathways. MK-2206 inhibits AKT phosphorylation. U0126 inhibits ERK phosphorylation. 5,15-DPP inhibits STAT3 dimerization and activation [21, 22]. We quantified changes in PDL-1 expression in cells treated with supernatants in the presence or absence of these inhibitors by densitometry of PDL-1 reactive bands in Western blots, normalized to the protein load of the individual lane of the SDS gel as determined by densitometry of GAPDH bands on the membrane. Figure 3a and b show Western blots of protein samples from BC-PAP or CAL-62 cells. Figure 3c depicts a quantification of protein bands by densitometry in both cell lines. Inhibition of STAT3 markedly reduced basal expression of PDL-1 and blocked up-regulation in BC-PAP as well as CAL-62 thyroid carcinoma cells stimulated with supernatant (Fig. 3c). In contrast, inhibition of ERK-phosphorylation blocked stimulation by supernatant but not basal expression of PDL-1 in BC-PAP-cells. In CAL-62-cells ERK-inhibition did reduce PDL-1 under neither one of the two conditions. Inhibition of PI3K-AKT by MK-2206 did not reduce PDL-1 expression in one of the cell lines under the conditions tested. Interestingly, sorafenib, an inhibitor of VEGF-receptor tyrosine and Raf kinase approved for treatment of thyroid cancer, stimulated PDL-1 expression in BC-PAP and CAL-62 cells (Fig. 3a, b).

In contrast, the tyrosine kinase inhibitors sunitinib, everolimus, and alpelisib [23] did not modulate PDL-1 expression in neither one of the cell lines (data not shown).

**Fig. 4:**
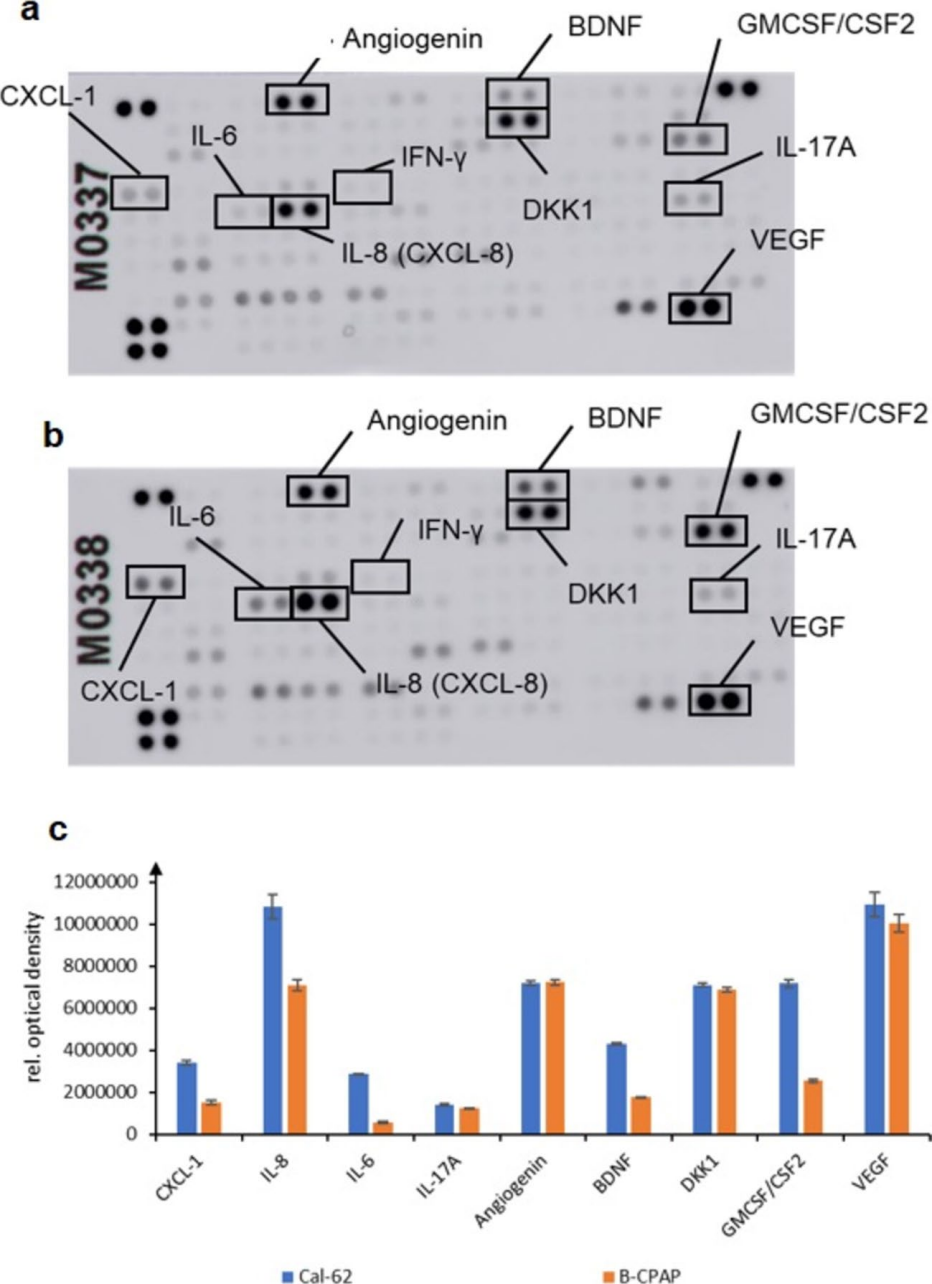
Cytokines in supernatants from papillary or anaplastic thyroid carcinoma. Cytokine ELISA arrays exposed to supernatants from thyroid carcinoma cells are shown. **a** B-CPAP-cells. **b** CAL-62-cells. **c** Densitometric analysis of cytokine concentrations in supernatants. Bars represent mean ± standard deviation

Our data indicate that in supernatant of thyroid carcinoma cells factors promoting PDL-1 expression by activation of the AKT, ERK, and STAT3 signaling pathways are present. We used a Cytokine Array Kit to investigate the cytokines present in the supernatants from CAL-62 and BC-PAP cells by an ELISA array. Indeed, a high number of different cytokines are found in the supernatants, including interferon-γ, IL-8, IL-6, IL-17A, CXCL1, angiogenin, VEGF, and GMCSF. The secretion pattern of the two cell lines (anaplastic or papillary thyroid cancer) was generally similar (Fig. 4a, b).

**Fig. 5:**
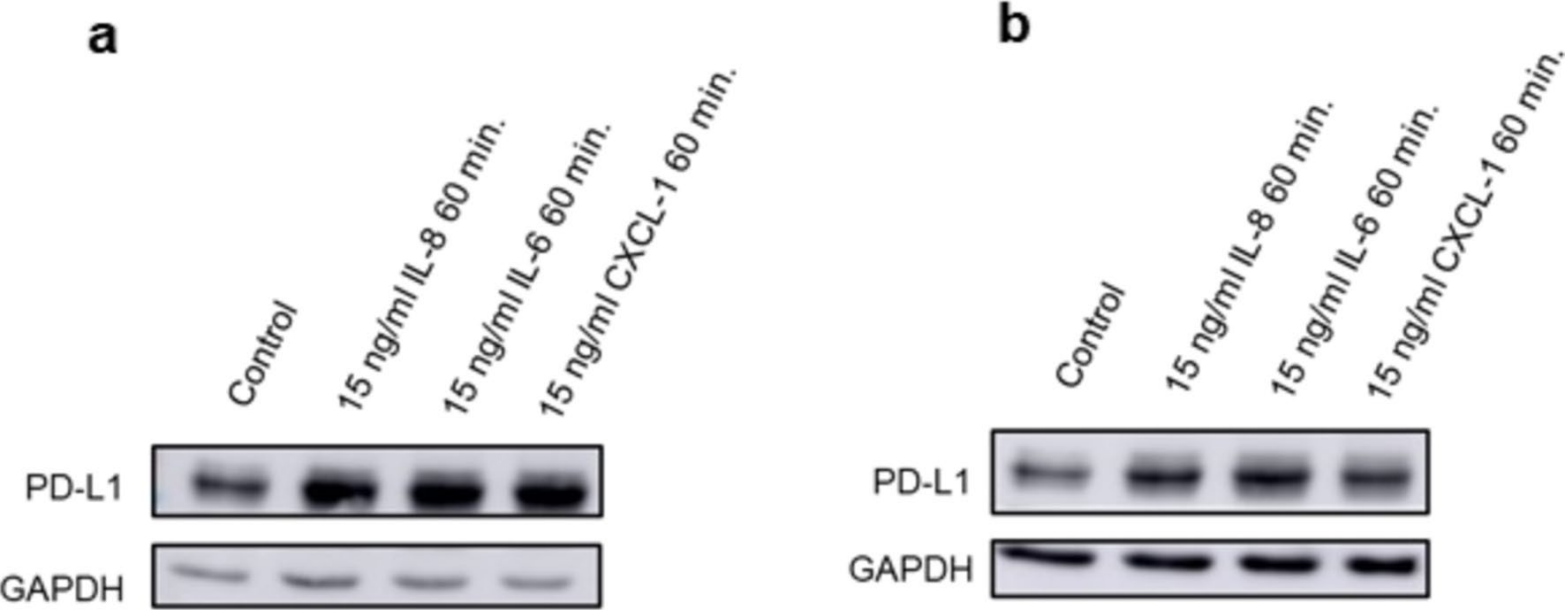
PDL-1 upregulations by IL-6, IL-8, and CXCL-1. Cells were stimulated with cytokines presents in supernatant from thyroid cells for the indicated time periods. PDL-1 expression was determined by Western blot in **a** B-CPAP and **b** CAL-62 cells. Blots are representative of three independent experiments

We asked whether the cytokines present in the supernatants individually induce PDL-1 expression in thyroid carcinoma cells. Therefore, we incubated BC-PAP or CAL-62 cells with IL-8, IL-6, and CXCL1 and determined PDL-1 expression by Western blot. We used cytokine concentrations as reported recently to stimulate PDL-1 expression [9]. Each of these cytokines induced PDL-1 in both cell lines (Fig. 5a, b).

It is widely accepted that interferon-γ secreted by immune cells is most important cytokine stimulating PDL-1 [22]. We found that a small amount of interferon-γ is also present in supernatant of thyroid carcinoma cells (the faint dots on the ELISA array membrane are marked with a black box in Fig. 4). It is also widely accepted that interferon-γ regulates PDL-1 primarily by the STAT3 pathway. We therefore tested whether interferon-γ induces PDL-1 also in thyroid carcinoma cells and whether the JAK-STAT pathway is involved. Indeed, interferon-γ induced PDL-1 expression and phosphorylation, i.e., activation of STAT-3. 5,15-DPP an inhibitor of STAT-3 blocked interferon-induced PDL-1 expression, as expected (data not shown) [22–24].

**Fig. 6:**
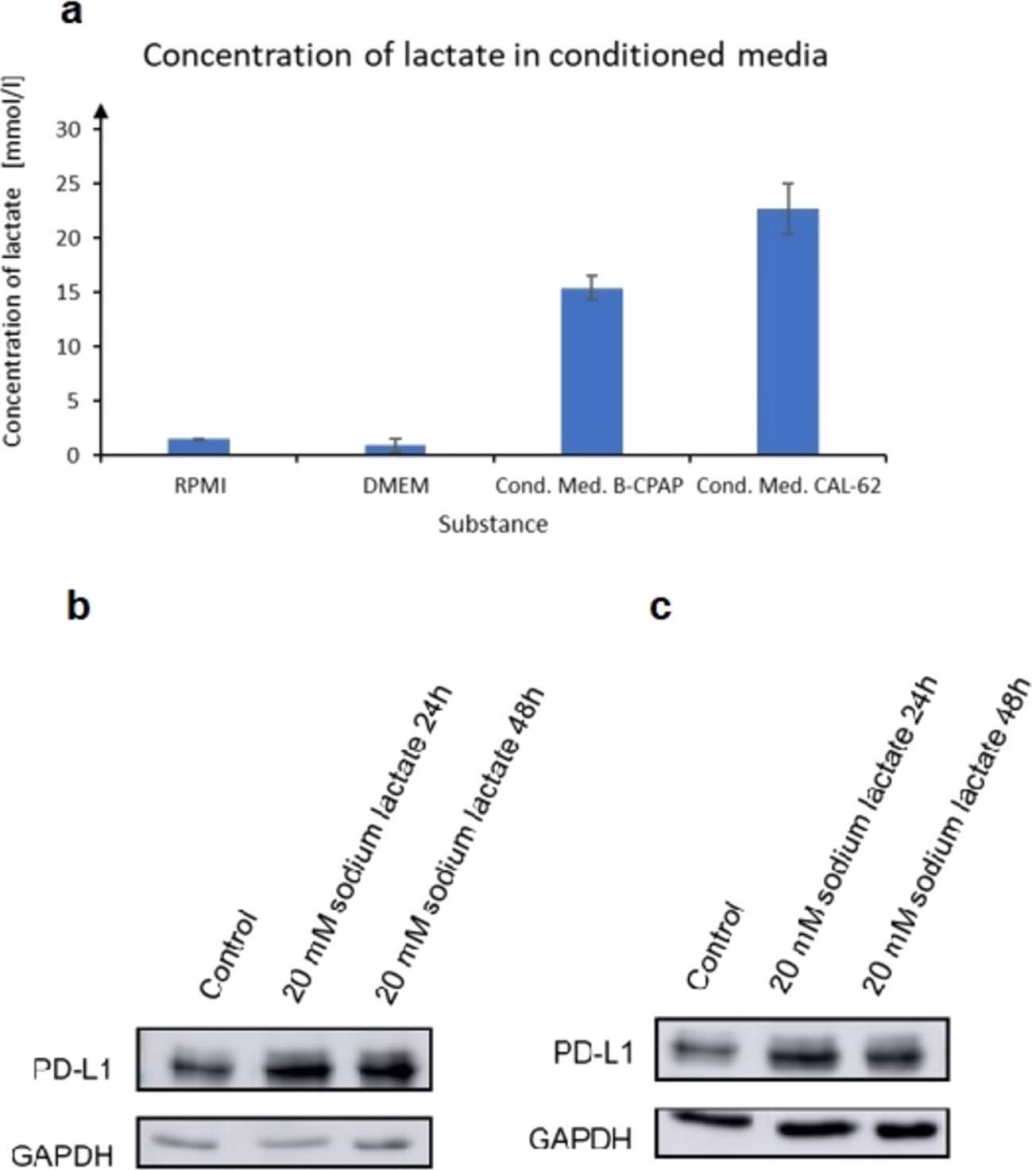
Modulation of PDL-1 expression by sodium lactate. **a** Lactate concentrations in supernatants from CAL-62 and BCPAP cells were measured as described. Values [mmol/l] ± SD are shown. Cells were grown with or without lactate for the indicated time periods. PDL-1 expression was determined by Western blot in **b** BC-PAP and **c** CAL-62 cells

Thyroid cancer cells secrete specific cytokines into their environment and thereby promote the expression of PDL-1. In addition to these specific mediators, unspecific changes such as reduced availability of nutrients due to growth, hypoxia, and secretion of metabolic products such as lactate may contribute to increased PDL-1 expression [25, 26]. We therefore studied whether lactate concentrations rising due to metabolism of tumor cells over time play a role. As expected, we found increasing lactate concentrations in the supernatants of thyroid cancer cells increasing with time (Fig. 6a). Moreover, incubation of cells with fresh culture media containing lactate increased PDL-1 expression in both cell lines (Fig. 6b, c).

**Fig. 7:**
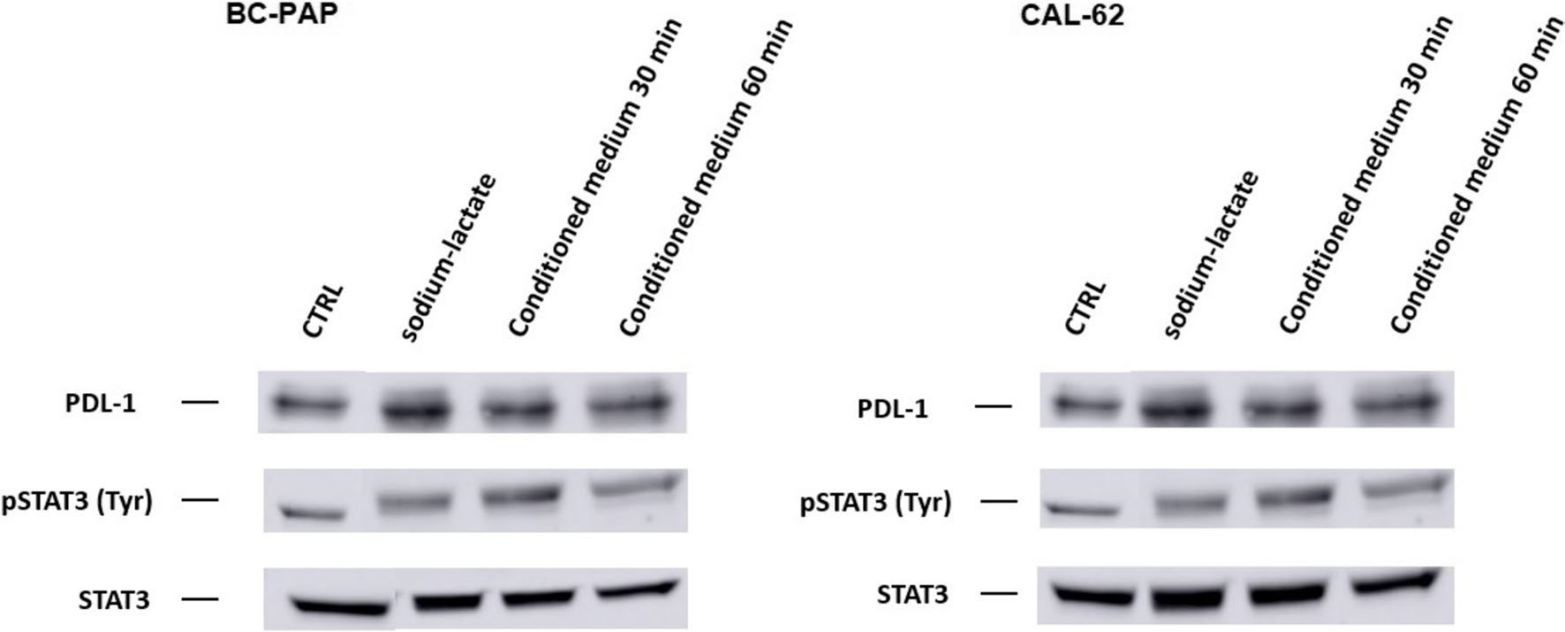
CAL-62 or BC-PAP-cells were stimulated with sodium lactate (24 h) or conditioned medium (30 or 60 min). PDL-1 and STAT3 expression as well as STAT3 phosphorylation were determined by Western blot. We found PDL-1-upregulation in parallel with STAT3 phosphorylation upon stimulation with sodium lactate or conditioned medium

Next, we tested whether conditioned medium or lactate increase STAT3-phosphorylation along with PDL-1 upregulation. As indicated by Fig. 7, lactate as wells as conditioned medium induced STAT3-phosporylation in parallel with PDL-1-upregulation in BC-PAP as well as in CAL-62 cells, consistent with the idea that factors are secreted by thyroid carcinoma cells up-regulating PDL-1 by STAT3-phosphorylation/activation.

## Discussion

We analyzed PDL-1 expression in cell lines from papillary and anaplastic thyroid cancer. In unstimulated BC-PAP and CAL-62 cells PDL-1 mRNA and protein was present. Stimulation of cells with supernatant from thyroid carcinoma cells induced upregulation of PDL-1 protein, suggesting an autocrine mechanism of upregulation. Indeed, using an ELISA array of cytokines, we found that a number of cytokines are present in the supernatants of both cell types. For example, we found high concentrations of IL-6, IL-8, γ-interferon, angiogenin, and CXCL1. Stimulation of cells with each of these cytokines tested increased protein expression of PDL-1, indicating that thyroid cancer cells produce a variety of cytokines up-regulating PDL-1 potentially contributing to immune escape of tumors. Interestingly, there was no significant difference in secretion pattern or concentration of cytokines in the supernatants of papillary and anaplastic cancer cells. Up-regulation of PDL-1 was not only due to secretion of cytokines but also due to formation of lactate by the cells, similar to hypoxic tumors with a high rate of anaerobic glycolysis. We also investigated the signal pathways regulating basal expression and upregulation of PDL-1. In both cell types, basal and stimulated PDL-1 levels were dependent on STAT3 activity. This is not surprising as most cytokines induce transcription via the JAK-STAT pathway [27]. On the other hand, in papillary thyroid cancer cells inhibition of ERK blocked upregulation of PDL-1 by supernatant, indicating crosstalk between MAP-kinase/ERK and STAT3 pathways in this type of thyroid cancer. This finding is in agreement with the high ERK/MAP-kinase activity present in most papillary thyroid carcinomas [1]. The most likely mechanism of crosstalk is phosphorylation of Ser727 in STAT3 [11, 28]. In anaplastic thyroid carcinoma cells, we found no influence of ERK inhibition on PDL-1 expression. It is remarkable that multiple cytokines individually capable of inducing PDL-1 expression are present in the supernatant of thyroid carcinoma cells, indicating that this may be an important and frequently utilized mechanism. A limitation of the data is that we used chemical inhibitors of the signal pathways studied potentially also blocking other signaling molecules regulating PDL-1.

Our data could be important for understanding of progression of thyroid cancer and for development of novel therapeutic strategies. The thyroid is subject to pathological immunological processes such as Hashimoto thyroiditis (HT) associated with an increased risk of cancer [29]. In HT patients, an immune reaction against thyroid cells leads to destruction of thyroid tissue driven by cytotoxic T and NK cells and cytokines such as interferon-γ. The source of pro-inflammatory cytokines in HT are immune cells as well as thyroid cells. This immune reaction resembles the T-cell-mediated tumor control [30]. As the initial trigger of HT is still under investigation at least in some patients, malignant transformation of single thyroid cells may activate T-cell response. Upregulation of checkpoints such as PDL-1 by the cytokines secreted in context of this immune response may promote malignant growth. Moreover, increasing TSH levels lead to activation of STAT3 in thyroid cells. Therefore, it is possible that in HT, destruction of thyroid tissue causes increasing TSH levels and subsequently STAT3-activation promoting cytokine secretion by thyroid and thyroid cancer cells promoting PDL-1 expression and immune escape. Regardless of their importance in HT, these mechanisms may contribute to progression of thyroid cancer. It is remarkable that our data suggest crosstalk between the ERK/MAP-kinase pathway and STAT3 in regulation of PDL-1. Therefore, it is possible that in papillary carcinoma with high ERK activity due to BRAFV600 mutation this pathway further enhances PDL-1 expression by Ser727 phosphorylation of STAT3 [12, 31].

It may also be possible to modulate PDL-1 expression by intervention with anti-cancer agents. We found that Sorafenib a tyrosine kinase inhibitor used in iodine-negative thyroid cancer upregulates PDL-1 in CAL-62 and BC-PAP cells. It is unclear whether this upregulation occurs in vivo and whether it contributes to Sorafenib resistance by reducing immune response. On the other hand, radiation of cells did not induce PDL-1 in the two cell lines (data not shown). STAT3 inhibitors reducing PDL-1 expression may also be an option for treatment in early thyroid cancer enhancing immune control of the tumors.

The cytokines found in the supernatant of BC-PAP and CAL-62 cells may have effects in biology of thyroid cancer beyond stimulation of PDL-1. CXCL1 stimulates proliferation, invasiveness and proliferation of thyroid cancer cells. IL-8 plays an important role for the growth of many tumor types stimulating angiogenesis, invasiveness, as well as metastatic potential [32]. Interestingly, IL-8 activates the Ras/MAP-Kinase/ERK pathway playing an important role in malignant transformation of thyroid cells as described above. Interferon-γ in secreted by thyroid carcinoma cells may exert growth promoting and inhibiting effects [12, 29].

Taken together, our data suggests that thyroid carcinoma create an environment by secretion of cytokines and later on under hypoxic conditions by formation of lactate promoting PDL-1 expression and immune escape.

## Data Availability

No datasets were generated or analysed during the current study.

## References

[1] Fagin J, Wells Samuel A. Biologic and clinical perspectives on thyroid cancer. N Engl J Med. 2016;375(23):2306–7. 10.1056/NEJMc1613118. 10.1056/NEJMc1613118 27959677

[2] Naoum GE, Morkos M, Kim B, Arafat W. Novel targeted therapies and immunotherapy for advanced thyroid cancers. Mol Cancer. 2018;17(1):S. 51. 10.1186/s12943-018-0786-0. 10.1186/s12943-018-0786-0 PMC5817719 29455653

[3] Bonnefond S, Davies T. Thyroid cancer—risks and causes. Oncol Hematol Rev. 2014;102:144–51. 10.3389/fendo.2014.00114.

[4] Strickler JH, Hanks BA, Khasraw M. Tumor mutational burden as a predictor of immunotherapy response: is more always better? In: Clin Cancer Res: an official journal of the American Association for Cancer Research. 2021;27(5):S. 1236-41. 10.1158/1078-0432.CCR-20-3054. 10.1158/1078-0432.CCR-20-3054 PMC9912042 33199494

[5] Menicali E, Guzzetti M, Morelli S, Moretti S, Puxeddu E. Immune landscape of thyroid cancers: new insights. Front Endocrinol. 2020;11:S. 637826. 10.3389/fendo.2020.637826. 10.3389/fendo.2020.637826 PMC8112200 33986723

[6] Moretti S, Menicali E, Nucci N, Guzzetti M, Morelli S, Puxeddu E. Therapy of endocrine disease Immunotherapy of advanced thyroid cancer: from bench to bedside. Euro J Endocrinol. 2020;183(2):R41–55. 10.1530/EJE-20-0283. 10.1530/EJE-20-0283 32449696

[7] Chowdhury S, Veyhl J, Jessa F, Polyakova O, Alenzi A, MacMillan C, et al. Programmed death-ligand 1 overexpression is a prognostic marker for aggressive papillary thyroid cancer and its variants. Oncotarget. 2016;7(22):S. 32318-32328. 10.18632/oncotarget.8698. 10.18632/oncotarget.8698 PMC5078015 27086918

[8] Dhatchinamoorthy K, Colbert JD, Rock KL. Cancer immune evasion through loss of MHC class I antigen presentation. Front Immunol. 2021;12:S. 636568. 10.3389/fimmu.2021.636568. 33767702 10.3389/fimmu.2021.636568 PMC7986854

[9] Sun L, Wang Q, Chen B, Zhao Y, Shen B, Wang H, et al. Gastric cancer mesenchymal stem cells derived IL-8 induces PD-L1-Expression in gastric cancer cells via STAT3/mTOR-c-Myc signal axis. Cell Death Dis. 2018;9:1–11. 10.1038/s41419-018-0988-9. 30206229 10.1038/s41419-018-0988-9 PMC6134105

[10] Shi R-L, Qu N, Luo T-X, Xiang J, Liao T, Sun G-H, et al. Programmed death-ligand 1 expression in papillary thyroid cancer and its correlation with clinicopathologic factors and recurrence. Thyroid: official journal of the American Thyroid Association. 2017;27(4):S. 537-45. 10.1089/thy.2016.0228. 27825291 10.1089/thy.2016.0228

[11] Thomas SJ, Snowden JA, Zeidler MP, Danson SJ. The role of JAK/STAT signalling in the pathogenesis, prognosis and treatment of solid tumours. Br J Cancer. 2015;113:365–71. 10.1038/bjc.2015.233. 26151455 10.1038/bjc.2015.233 PMC4522639

[12] Sosonkina N, Starenki D, Park JI. The role of STAT3 in thyroid cancer. Cancers. 2014;6:526–44. 10.3390/cancers6010526. 24662939 10.3390/cancers6010526 PMC3980610

[13] Cameselle-García S, Abdulkader-Sande S, Sánchez-Ares M, Rodríguez-Carnero G, Garcia-Gómez J, Gude-Sampedro F, et al. PD-L1 expression and immune cells in anaplastic carcinoma and poorly differentiated carcinoma of the human thyroid gland: a retrospective study. In: Oncology letters. 2021;22(1):S. 553. 10.3892/ol.2021.12814. 10.3892/ol.2021.12814 PMC8170268 34093774

[14] Bi Y, Ren X, Bai X, Meng Y, Luo Y, Cao J, et al. PD-1/PD-L1 expressions in medullary thyroid carcinoma: clinicopathologic and prognostic analysis of Chinese population. In: Euro J Surgical Oncol : the journal of the European Society of Surgical Oncology and the British Association of Surgical Oncology. 2019;45(3):S. 353-8. 10.1016/j.ejso.2018.10.060. 10.1016/j.ejso.2018.10.060 30528043

[15] Bongiovanni M, Rebecchini C, Saglietti C, Bulliard J-L, Marino L, de Leval L, Sykiotis GP. Very low expression of PD-L1 in medullary thyroid carcinoma. In: Endocrine-related cancer. 2017;24(6):L35–8. 10.1530/ERC-17-0104. 10.1530/ERC-17-0104 PMC5457503 28420659

[16] Garcia-Alvarez A, Hernando J, Carmona-Alonso A, Capdevila J. What is the status of immunotherapy in thyroid neoplasms? In: Frontiers in endocrinology. 2022;13:S. 929091. 10.3389/fendo.2022.929091. 10.3389/fendo.2022.929091 PMC9389039 35992118

[17] Barin JG, Afanasyeva M, Talor MV, Rose NR, Burek CL, Caturegli P. Thyroid-specific expression of IFN-gamma limits experimental autoimmune thyroiditis by suppressing lymphocyte activation in cervical lymph nodes. J Immunol Baltim Md. 1950;2003(170):5523–9. 10.4049/jimmunol.170.11.5523. 10.4049/jimmunol.170.11.5523 12759429

[18] Bauerle KT, Schweppe RE, Lund G, Kotnis G, Deep G, Agarwal R, et al. Nuclear factor κB–dependent regulation of angiogenesis, and metastasis in an in vivo model of thyroid cancer is associated with secreted interleukin-8. J Clin Endocrinol Metab. 2014;99:1436–44. 10.1210/jc.2013-3636. 10.1210/jc.2013-3636 PMC4121024 24758177

[19] Ju X, Zhang H, Zhou Z, Wang Q. Regulation of PD-L1-Expression in cancer and clinical implications in immunotherapy. Am J Cancer Res 2020; 10: 1–11. www.ajcr.us /ISSN:2156–6976/ajcr0106488. PMC7017746 32064150

[20] Stassi G, Todaro M, Zerilli M, Ricci-Vitiani L, Di Liberto D, Patti M, et al. Thyroid cancer resistance to chemotherapeutic drugs via autocrine production of interleukin-4 and interleukin-10. Cancer Res. 2003;63:6784–90. 14583474

[21] Uehara Y, Mochizuki M, Matsuno K, et al. Novel high-throughput screening system for identifying STAT3–SH2 antagonists. J Biochem Biophysical Res Commun. 2009;380:627–31. 10.1016/j.bbrc.2009.01.137. 10.1016/j.bbrc.2009.01.137 19285012

[22] Hirai H, Sootome Y, Nakatsuru Y, et al. MK-2206, an allosteric Akt inhibitor, enhances antitumor efficacy by standard chemotherapeutic agents or molecular targeted drugs in vitro and in vivo. Mol Cancer Ther. 2010;9:1956–67. 10.1158/1535-7163.MCT-09-1012. 20571069 10.1158/1535-7163.MCT-09-1012

[23] Marotta V, Sciammarella C, Vitale M, et al. The evolving field of kinase inhibitors in thyroid cancer. Crit Rev Oncol Hematol. 2015;93:60–73. 10.1016/j.critrevonc.2014.08.007. 25240824 10.1016/j.critrevonc.2014.08.007

[24] Zhao T, Li Y, Zhang J, Zhang B. PD-L1-Expression increased by IFN-γ via JAK2-STAT1 signaling and predicts a poor survival in colorectal cancer. Oncol Lett. 2020;20:1127–34. 10.3892/ol.2020.11647. 32724352 10.3892/ol.2020.11647 PMC7377091

[25] Verma AK, Messerli SM, Miskimins WK. Lactate induces PD-L1 in HRASG12V-positive oropharyngeal squamous cell carcinoma. Oncotarget. 2020;11:1493–504. 10.18632/oncotarget.27348. 32391119 10.18632/oncotarget.27348 PMC7197448

[26] Yabu M, Shime H, Hara H, Saito T, Matsumoto M, Seya T, et al. IL-23-dependent and -independent enhancement pathways of IL-17A production by lactic acid. Int Immunol. 2011;23:29–41. 10.1093/intimm/dxq455. 21131367 10.1093/intimm/dxq455

[27] Lv Y, Mi P, Babon JM, Kobe FG, et al. Small molecule drug discovery targeting the JAK-STAT pathway. Pharmacol Res. 2024;304:107217. 10.1016/j.phrs.2024.107217. 10.1016/j.phrs.2024.107217 38777110

[28] Lin WH, Chang YW, Hong MX, et al. STAT3 phosphorylation at Ser727 and Tyr705 differentially regulates the EMT-MET switch and cancer metastasis. Oncogene. 2021;40:791–805. 10.1038/s41388-020-01566-8. 33262462 10.1038/s41388-020-01566-8 PMC7843420

[29] Mansoor A-H, Magtooph M. The role of Interferon (IFN- γ) in thyroid autoimmunity. J Coll Educ Pure Sci. 2019;9:221–6. 10.32792/utq.jceps.09.01.23.

[30] Ehlers M, Schott M. Hashimoto’s thyroiditis and papillary thyroid cancer: are they immunologically linked? Trends Endocrinol Metab. 2014;25:656–64. 10.1016/j.tem.2014.09.001. 25306886 10.1016/j.tem.2014.09.001

[31] Shinohara M, Chung YJ, Saji M, Ringel MD. AKT in thyroid tumorigenesis and progression. Endocrinology. 2007;148:942–7. 10.1210/en.2006-0937. 16946008 10.1210/en.2006-0937

[32] Rotondi M, Coperchini F, Latrofa F, Chiovato L. Role of chemokines in thyroid cancer microenvironment: is CXCL8 the main player? Front Endocrinol 21. 2018;9:314. 10.3389/fendo.2018.00314. 10.3389/fendo.2018.00314 PMC6021500 29977225

